# Comparative Analysis of Shapley Values Enhances Transcriptomics Insights across Some Common Uterine Pathologies

**DOI:** 10.3390/genes15060723

**Published:** 2024-06-01

**Authors:** José A. Castro-Martínez, Eva Vargas, Leticia Díaz-Beltrán, Francisco J. Esteban

**Affiliations:** 1 Systems Biology Unit, Department of Experimental Biology, Faculty of Experimental Sciences, University of Jaén, 23071 Jaén, Spain; j.a.castrobiologo@gmail.com (J.A.C.-M.); evargas@ujaen.es (E.V.); ldiaz@fibao.es (L.D.-B.); 2 Clinical Research Unit, Department of Medical Oncology, University Hospital of Jaén, 23007 Jaén, Spain

**Keywords:** endometrial cancer, endometriosis, microarrays, systems biology, transcriptomics, uterine leiomyomas

## Abstract

Uterine pathologies pose a challenge to women’s health on a global scale. Despite extensive research, the causes and origin of some of these common disorders are not well defined yet. This study presents a comprehensive analysis of transcriptome data from diverse datasets encompassing relevant uterine pathologies such as endometriosis, endometrial cancer and uterine leiomyomas. Leveraging the Comparative Analysis of Shapley values (CASh) technique, we demonstrate its efficacy in improving the outcomes of the classical differential expression analysis on transcriptomic data derived from microarray experiments. CASh integrates the microarray game algorithm with Bootstrap resampling, offering a robust statistical framework to mitigate the impact of potential outliers in the expression data. Our findings unveil novel insights into the molecular signatures underlying these gynecological disorders, highlighting CASh as a valuable tool for enhancing the precision of transcriptomics analyses in complex biological contexts. This research contributes to a deeper understanding of gene expression patterns and potential biomarkers associated with these pathologies, offering implications for future diagnostic and therapeutic strategies.

## 1. Introduction

Disorders affecting the uterus represent significant burdens on women’s health worldwide. These conditions, characterized by aberrant cellular proliferation and tissue growth within the uterine environment, manifest with diverse clinical presentations and pose substantial challenges in diagnosis and management [1].

Endometriosis is a chronic and debilitating gynecological disorder where endometrial-like tissue grows outside the uterine cavity, often affecting the ovaries, fallopian tubes, and pelvic peritoneum, with possible distant sites like the lungs and bowel [2]. It impacts about 10% of women in their reproductive years, and potentially more due to undiagnosed cases [3]. The main symptom of endometriosis is pelvic pain, linked to the menstrual cycle and manifesting as painful periods, pain during intercourse, and chronic pelvic pain, which can severely impair daily activities [4]. Additionally, 30–50% of affected women experience infertility. Other symptoms include menstrual irregularities, painful bowel movements, urinary issues, and fatigue. The etiology of endometriosis involves genetic, immunological, and environmental factors, contributing to its complex and varied presentation [5]. Treatment typically requires a multidisciplinary approach, including hormonal therapies and surgery, aimed at relieving symptoms, preventing progression, and preserving fertility [6]. However, recurrence is common. Given its significant impact on life quality and health, there is a crucial need for better diagnostic and therapeutic strategies to enhance outcomes for those women suffering from this pervasive condition.

Uterine leiomyomas, often known as fibroids, are benign smooth muscle tumors that arise within the uterine wall, affecting up to 70% of women by age 50 [7]. These tumors can cause a range of symptoms, including abnormal uterine bleeding, pelvic pressure, frequent urination, constipation, and reproductive issues such as difficulty conceiving and complications during pregnancy [8]. The growth of fibroids is influenced by hormonal factors, particularly estrogen and progesterone, and is more prevalent and severe in women of African descent [9,10]. Treatment varies from pharmacological management to regulate hormones and alleviate symptoms to surgical options like myomectomy and hysterectomy, depending on the severity and the reproductive goals of the patient. Recent advances in less invasive techniques and ongoing genetic research promise better, personalized treatments to mitigate the impact of fibroids on women’s health.

Endometrial cancer, which develops from the malignant transformation of the endometrial lining, is recognized as the most prevalent gynecologic malignancy in developed countries and its incidence is on the rise worldwide [11]. This type of cancer is typically diagnosed in postmenopausal women, although rates among younger women are increasing, which may be attributed to rising obesity rates, one of the key risk factors [12]. The disease manifests through symptoms like abnormal bleeding, pelvic pain, and weight loss. Early detection through symptoms awareness and routine screening in at-risk populations is crucial for effective treatment [13]. Treatment strategies commonly involve a combination of surgery, such as hysterectomy, followed by radiation or chemotherapy depending on the stage and grade of the tumor [11,14]. Advances in molecular profiling have begun to highlight the genetic underpinnings of the disease, offering the potential for targeted therapies that could improve prognosis and tailor treatments to individual patient profiles, enhancing outcomes and potentially reducing side effects.

Despite the high prevalence of these common gynecological conditions and the ongoing debate about the existence of a genetic overlap and comorbidity among them, the molecular basis of these pathologies has yet to be determined [1,15]. Thus, understanding the molecular mechanisms underlying uterine pathologies is crucial for the development of targeted therapeutic interventions and improved patient outcomes [16].

Advances in omics technologies, particularly in microarray analyses, have paved the way to the comprehensive exploration of gene expression patterns associated with uterine conditions [17,18,19,20,21,22,23]. Microarray technologies provide the measurement of the expression levels for thousands of genes at a glance, which allows to obtain a deeper insight into the dysregulated molecular pathways implicated in the pathogenesis of several diseases [24,25,26,27,28]. The identification of differentially expressed genes (DEGs) represents a keystone in microarray data analysis. Traditionally, these methods rank genes based on individual *p*-values; however, these *p*-values do not always correlate with biologically significant signals. In some instances, very small *p*-values, which suggest high significance, may not be relevant to the biological condition being studied, while larger *p*-values, often dismissed as insignificant, might be associated with genes critical for certain biological mechanisms [29]. Classical approaches for microarray data analyses usually apply Welch’s *t*-test and linear-model-based methods such as Empirical Bayes as statistical methods for the identification of DEGs by comparing expression levels between two experimental groups or conditions [30,31]. However, these traditional methods may overlook significant changes at the gene expression level, especially in complex diseases such as those affecting the uterus, which possess heterogeneous molecular profiles [32,33].

To overcome the shortcomings associated with *p*-value-based approaches, which often include the excessive suppression of biologically pertinent signals by multiple testing correction methods, more robust methodologies have been developed [29,34,35]. Notably, one such method incorporates game theory, utilizing a computational index known as the Shapley value [29]. This approach provides a more nuanced assessment of gene significance by evaluating the cumulative contribution of each gene within the context of the entire gene set analyzed. The Shapley value quantifies the importance of each gene by considering its contribution in conjunction with the contributions of all other genes in the same experiment [36]. This approach, which integrates game theory with traditional statistical analyses, offers a powerful tool for enhancing the detection and interpretation of meaningful gene expression differences [29].

In this sense, here, we integrated the methodology of microarray games, specifically leveraging Shapley values, to analyze gene expression data related to various uterine pathologies. This approach employs game theory to enhance the detection and functional analysis of genes implicated in complex biological conditions, such as autism spectrum disorder (ASD) [29]. Through this technique, which considers the average marginal contribution of each gene within all possible coalitions, we anticipate revealing critical insights into the genetic basis of these diseases, potentially leading to novel diagnostic and therapeutic strategies. This game-theoretic approach not only increases the power to identify key genetic players but also enriches our understanding of their biological roles in complex multi-genic pathologies.

Thus, in the present study, we aim to investigate the gene expression profiles associated with three of the most common uterine pathologies through the application of two different methods for the microarray data analysis: (i) a conventional method using Welch’s *t*-test and Empirical Bayes methods, and (ii) a complementary analysis based on the Comparative Analysis of Shapley value (CASh) method derived from game theory, a method that, as commented above, we have previously demonstrated that significantly increases the power to identify DEGs [29].

## 2. Materials and Methods

### 2.1. Microarray Expression Data Acquisition, Processing, and Exploratory Analysis

Microarray data were obtained from Gene Expression Omnibus (GEO) database (https://www.ncbi.nlm.nih.gov/geo/, accessed on 28 May 2024). For the selection of datasets of interest, raw data from Affymetrix commercial microarrays Affymetrix Human Genome U133A Array (HG-U133A), Affymetrix Human Genome U133A 2.0 Array (HG-U133A_2), Affymetrix Human Genome U133 Plus 2.0 Array (HG-U133_Plus_2), and Affymetrix Human Gene 1.0 ST Array [transcript (gene) version] (HuGene-1_0-st), were accessed preferentially, when possible.

CEL files from two datasets of endometriosis (GSE7846, GSE17504) [37,38], two datasets of uterine leiomyomas (GSE12814, GSE23112) [39,40], and two datasets of endometrial cancer (GSE36389, GSE63678) [41] were retrieved from GEO repository. Raw data were downloaded for each dataset and preprocessing, quality control and normalization based on relative log expression (RLE), normalized unscaled standard error (NUSE), and Robust Multi-Array Average expression measure (RMA) methods were computed using ‘affy’ (version 1.82.0) and ‘affyPLM’ (version 1.80.0) packages in RStudio (version 2023.12.1 run under R 4.3.2) [42,43,44]. Finally, expression matrices were generated and samples were classified into experimental and control groups for further analyses (Supplementary Table S1).

Each dataset was processed independently in order to identify DEGs. To conduct differential expression analyses between patients and controls, two approaches were performed: (i) a conventional approach based on the utilization of Welch’s *t*-test and Empirical Bayes methods, and (ii) an alternative method rooted on CASh technique.

Exploratory techniques, commonly used in microarray data analysis, were applied to our datasets. We conducted Principal Component Analysis (PCA), heatmaps, and volcano plot analyses to provide a comprehensive evaluation of gene expression patterns. The PCA illustrates the distribution of the gene expression patterns at two levels: the whole set of genes in each dataset vs. differentially expressed genes after Comparative Analysis of Shapley value analysis (*p*-value < 0.01). Heatmaps were generated to show the differentially expressed genes identified through Empirical Bayes analysis (raw *p*-value < 0.05) and CASh analysis (*p*-value < 0.01), highlighting the clustering of samples based on disease status. Additionally, volcano plots were created to compare Empirical Bayes and CASh *p*-values. These plots provide a visual representation of the relationship between the test statistics of the different methods used for the detection of DEGs (please see Supplementary Figures S1–S3 for further detail).

#### 2.1.1. Classical Approaches

Conventional analyses for the detection of DEGs were performed using Welch’s *t*-test implemented in the ‘multtest’ (version 2.60.0) package in RStudio (version 2023.12.1 run under R 4.3.2) [45]. Since, in microarrays experiments, the number of replicates is usually small and the number of genes is usually very large (which makes multiple testing an extreme problem), the ordinary *t*-tests are known to suffer from low power and, therefore, are not considered the best option for filtering out regulated genes [46,47]. Most multiple testing adjustments are relatively conservative, especially when the number of replicates is small [47]. This common problem can be handled by Bayesian-based methods such as Empirical Bayes. Here, we applied the Empirical Bayes as implemented in the Bioconductor ‘limma’ R package (https://bioconductor.org/packages/release/bioc/html/limma.html, accessed on 28 May 2024).

The significant DEGs were detected after multiple testing correction using the Benjamini and Hochberg method to control for False Discovery Rate (FDR) [48]. A significance threshold of an adjusted *p*-value (FDR) < 0.05 or <0.01 was applied.

#### 2.1.2. Comparative Analysis of Shapley Value (CASh) Approach

We applied the Comparative Analysis of Shapley value (CASh) method to identify DEGs based on their co-operative contribution to overall gene expression changes [49]. The Shapley value, a concept derived from game theory, quantifies the marginal contribution of each gene to the collective expression change observed in the dataset [50]. CASh is a statistical technique that combines the microarray game algorithm (applied to transcriptomic values obtained from microarray experiments) with the Bootstrap technique, that applies random resampling of certain values, aiming to compensate for potential outliers in the data matrix [49,51,52,53]. Therefore, CASh considers gene expression as a co-operative game, where each gene contributes to the observed expression changes in a collaborative manner.

In this context, a co-operative game is defined by a set N of players (genes) and a characteristic function v that assigns a value to each subset of genes, representing their combined contribution. The Shapley value $\phi_{i}$ for a gene i is calculated as its average marginal contribution across all possible subsets of genes, providing a robust measure of each gene’s importance in the study.

Formally, given a coalitional game $\left(N,v\right)$, for each player i ∈N, the Shapley value $\phi \left(v\right)$ is defined by:

$$\phi_{i}=\frac{1}{n!}\sum_{\pi }\left[v\left(P\left(\pi ,i\right)\cup \{i\}\right)-v\left(P\left(\pi ,i\right)\right)\right],$$

where π is a permutation of players, $P\left(\pi ,i\right)$ is the set of players that precede player i in the permutation π, and n is the cardinality of N.

We refer to a boolean matrix (see below) $B\in \{0,1{\}}^{n\times k}$, where k≥1 is the number of arrays, and the boolean values 0–1 represent two complementary expression properties, for example, normal expression (coded by 0) and over-expression (coded by 1). Let B.j be the jth column of B; we define the support of B.j, denoted by $sp\left(B.j\right)$, as the set $sp\left(B.j\right)=\{i\in \{1,\ldots ,n\}:B_{ij}=1\}$. The microarray game corresponding to B is defined as the coalitional game $\left(N,w\right)$, where $w:2^{N}\to R^{+}$ is such that $w\left(T\right)$ is the rate of occurrences of coalition T as a winning coalition, i.e., as a superset of the supports in the boolean matrix B; in next formula, $w\left(T\right)$, for each $T\in 2^{N}\setminus \{\emptyset \}$, is defined as the value

$$w\left(T\right)=\frac{c\left(\Theta \left(T\right)\right)}{k},$$

where $c\left(\Theta \left(T\right)\right)$ is the cardinality of the set $\Theta \left(T\right)=\{j\in K:sp\left(B.j\right)\subseteq T,sp\left(B.j\right)\neq \emptyset \}$, with the set of arrays K={1, …, k} and $v\left(\emptyset \right)=0$. Since it is computationally too expensive to calculate the Shapley value $\phi \left(w\right)$ of game $\left(N,w\right)$ according to relation $\phi_{i}$, [49] introduced an easy way to calculate $\phi \left(w\right)$ for whatever microarray game $\left(N,w\right).$ We have adapted the scripts from these authors [49] run under R (https://www.r-project.org/, accessed on 28 May 2024).

In our study, CASh method was applied to the detection of DEGs using two levels of restriction by establishing 0.01 (more restrictive) and 0.05 (less restrictive) as cutoff *p*-values. These genes were processed to discriminate over-regulated or under-regulated levels based on standard deviations from the control. Boolean matrices were constructed to represent these expression states, which were then used to define microarray games and calculate the corresponding Shapley values.

A final matrix incorporating the expression levels of an arbitrary number of genes and samples was generated from the original data as previously described. The matrix included genes with a raw *p*-value of less than 0.01, or 0.05, and categorized the samples into distinct groups (e.g., patients with specific conditions and healthy controls). To differentiate over-regulated gene expression levels compared to controls, each continuous value in the gene expression vector was coded as 1 if it was equal to or greater than the mean plus the standard deviation of the control group expressions, and as 0 otherwise. This processing created a boolean matrix with values {0, 1} reflecting these conditions.

Separately, a similar approach was used to identify under-regulated gene expressions, where each value less than the mean minus the standard deviation of control expressions was coded as 1, with all other values coded as 0. This also resulted in a boolean matrix with rows corresponding to genes and columns to samples. These boolean matrices were then split according to the distinctions between the sample groups, forming separate matrices for each group. From these matrices, microarray games were defined for each condition, and Shapley values were calculated to assess the significance of each gene’s contribution to the conditions being studied.

To mitigate the impact of random high Shapley values, a resampling procedure was applied, similar to that described elsewhere [49]. Bootstrap resampling with 1000 iterations was computed in each analysis. This method, termed CASh, helps in refining the selection of genes significantly associated with the conditions under study.

To reduce the likelihood of detecting false positives, corrections for multiple testing were applied, and Shapley values were compared against statistically significant thresholds. In addition, Fold Changes (FC) was evaluated. Genes with *p*-values below 0.01 or 0.05 and |FC| > 2 were considered as statistically significant.

### 2.2. Gene Set Enrichment Analysis and Functional Annotation

In our study, we employed the g:Profiler functional profiling tool (version e111_eg58_p18_30541362), specifically, the g:GOSt module (https://biit.cs.ut.ee/gprofiler/gost, accessed on 28 May 2024), to conduct an extensive analysis of the biological processes and pathways influenced by differentially expressed genes (DEGs). This tool utilizes Gene Ontology (GO) terms to provide a rich, annotated landscape of gene functions and interactions [54,55]. Gene Ontology offers a structured vocabulary that can classify and integrate biological data across species based on three main categories: biological processes (BP), cellular components (CC), and molecular functions (MF). By inputting the list of DEGs into g:GOSt, the tool maps these genes to known GO terms, allowing us to identify which biological pathways and processes are enriched with these genes. This enrichment analysis helps in understanding the roles these DEGs may play in the specific conditions under study. The g:GOSt module performs its analysis by comparing the list of input genes against databases of known gene and protein functions, looking for statistically significant over-representations of specific functions or pathways. This is achieved through various statistical methods, including Fisher’s Exact Test, to calculate enrichment *p*-values, which help in discerning which processes or pathways are more involved with the set of DEGs than would be expected by chance. The results from g:GOSt not only highlight the predominant biological themes associated with the DEGs but also provide insights into the potential molecular mechanisms driving the disease or condition. For example, if a significant number of DEGs are involved in inflammatory response pathways, this could indicate that inflammation plays a crucial role in the pathology of the condition being studied. Furthermore, the outcomes from such analyses can guide experimental design by identifying key pathways that could be targeted for further experimental validation or therapeutic intervention. This makes tools like g:Profiler indispensable in the genomic era, allowing researchers to translate large datasets of gene expression information into actionable biological insights [54,55].

In the process of analyzing gene expression data, it is crucial to ensure that transcript identifiers (IDs) are accurately annotated and standardized to official gene symbols. This step is fundamental for consolidating data from different sources and facilitating meaningful biological interpretation. For this purpose, we utilized the g:Convert tool available on the g:Profiler webserver (https://biit.cs.ut.ee/gprofiler/convert, accessed on 28 May 2024). This tool is designed to convert various biological identifiers into recognized gene symbols, enhancing the consistency and reliability of genomic data analysis. The g:Convert module supports a wide range of biological identifiers, including but not limited to Ensembl IDs, UniProt IDs, RefSeq, and others, allowing researchers to input data from various experimental outputs and databases. Upon inputting transcript IDs into g:Convert, the tool maps these IDs to the official gene symbols based on the most up-to-date and comprehensive databases. This ensures that subsequent analyses, such as gene expression profiling or functional enrichment, are performed on verified and universally accepted nomenclature, thereby reducing the risk of errors and inconsistencies. In cases where transcript names are ambiguous, which can occur due to the presence of multiple identifiers for a single gene or due to updates in genomic databases, we prioritized IDs that have the most GO annotations. This approach is grounded in the rationale that identifiers with more extensive annotations are likely more researched and documented, thus offering a higher degree of reliability. By choosing IDs with the most GO annotations, we aimed to enhance the robustness of our dataset, ensuring that the functional analysis reflects well-supported gene functions and interactions. The use of the g:Convert tool in this manner not only streamlines the process of gene annotation but also significantly enhances the quality of the data being analyzed. Accurate annotation is critical as it directly impacts the interpretation of the biological data and the conclusions drawn from research studies. As gene databases are continually updated and refined, tools like g:Convert are invaluable for maintaining the accuracy and relevance of genomic research, providing researchers with confidence in their analytical outputs [54,55].

Here, we conducted an in-depth analysis of GO categories, which serve to categorize gene products based on their involvement in BP, CC, and MF. To determine the significance of these GO categories, FDR was applied, requiring that GO terms exhibit an FDR value below 0.05 to be considered significantly enriched. This stringent threshold ensures that only the most robust associations are identified, minimizing the likelihood of false positives. Subsequently, the top ten significantly enriched GO terms within each category were identified for further investigation. To visually represent the findings, top ten significantly enriched GO terms in each category were plotted for CASh 0.05 comparisons using ‘ggplot2’ (version 3.5.1) RStudio package [56].

## 3. Results

### 3.1. Datasets and Samples Analyzed

Gene expression data from six datasets covering a total of 68 samples were accessed. Table 1 describes the main characteristics of the datasets included in our study.

Datasets were analyzed for the detection of DEGs using two different strategies. First, the use of conventional methods based on Welch’s *t*-test and Empirical Bayes was applied. Then, an alternative analysis based on the CASh method was performed. The use of Welch’s *t*-test and Empirical Bayes did not allow us, in general, to identify any DEGs, while several transcripts were revealed when using the CASh method with both 0.01 and 0.05 cutoff raw *p*-values for the preselection of DEGs (Table 2). The total lists of DEGs for each dataset detected after each comparison are shown in Supplementary Table S2. Our analyses revealed that the application of the CASh method allows a better detection of differentially expressed genes in the six datasets analyzed.

### 3.2. Functional Enrichment Analysis of the Differentially Expressed Genes

**Figure 1:**
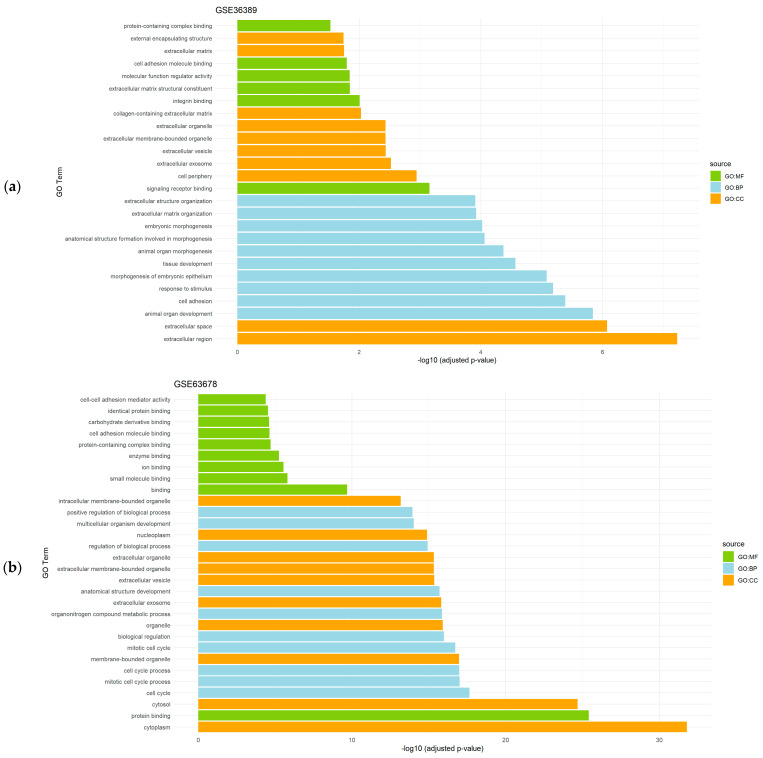
Gene set enrichment analysis results showing the significantly enriched Gene Ontology (GO) terms of the differentially expressed genes in endometrial cancer datasets: (**a**) GSE36389 dataset; and (**b**) GSE63678 dataset. For each dataset, significantly enriched molecular functions (GO:MF), biological processes (GO:BP), and cellular components (GO:CC) are shown.

Given the restrictive criteria applied when running the CASh 0.01 method and FDR correction of *p*-values, the number of DEGs detected did not allow us to obtain a number of significantly enriched pathways associated to some gene sets. However, a functional enrichment analysis of the differentially expressed genes obtained after the application of the CASh 0.05 method revealed relevant significantly enriched processes in the analyzed datasets. In endometrial cancer datasets (GSE36389 and GSE63678), DEGs were mainly related to BP such as development and morphogenesis, CC and MF were mainly associated with extracellular locations and diverse molecules binding, respectively (Figure 1).

**Figure 2:**
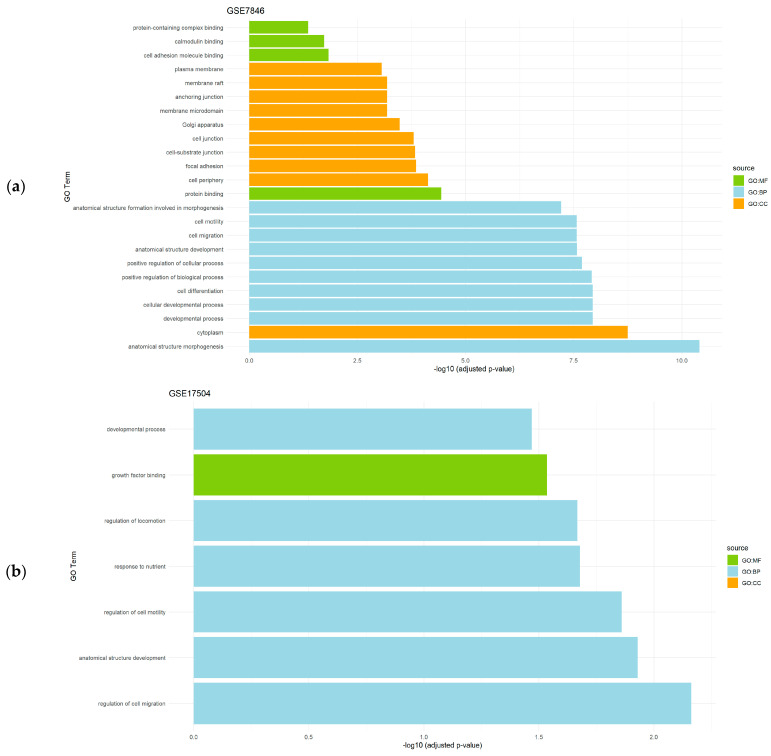
Gene set enrichment analysis results showing the significantly enriched Gene Ontology (GO) terms of the differentially expressed genes in endometriosis datasets: (**a**) GSE7846 dataset; and (**b**) GSE17504 dataset. For each dataset, significantly enriched molecular functions (GO:MF), biological processes (GO:BP), and cellular components (GO:CC) are shown.

Regarding datasets of endometriosis (GSE7846 and GSE17504), the top significantly enriched BP were related to development, the regulation of several cellular processes, and morphogenesis. The CC results revealed cytoplasm and cell periphery to be significantly relevant, and the MF analysis detected functions mainly associated to protein activity (Figure 2).

**Figure 3:**
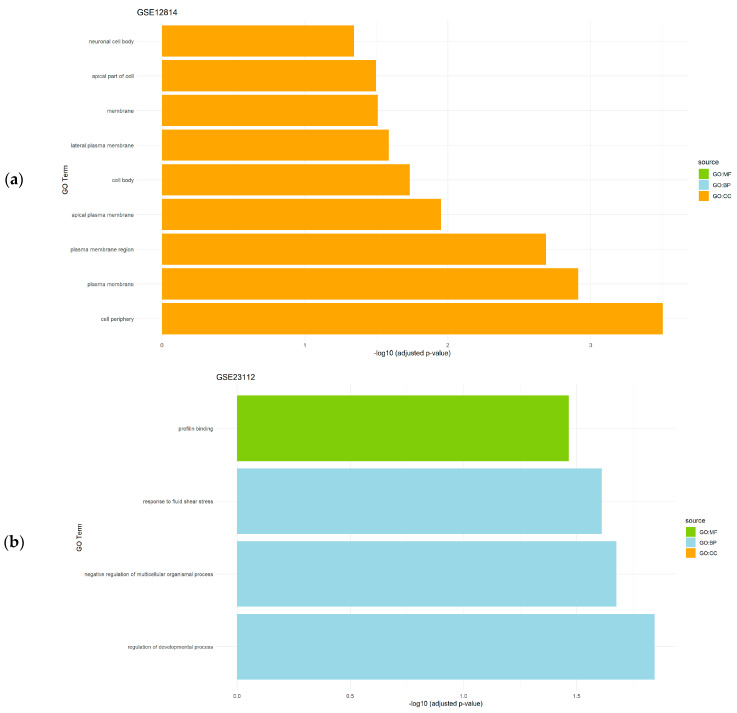
Gene set enrichment analysis results showing the significantly enriched Gene Ontology (GO) terms of the differentially expressed genes in uterine leiomyoma datasets: (**a**) GSE12814 dataset; and (**b**) GSE23112 dataset. For each dataset, significantly enriched molecular functions (GO:MF), biological processes (GO:BP), and cellular components (GO:CC) are shown.

The gene set enrichment analysis of the differentially expressed genes obtained after the application of the CASh 0.05 method in uterine leiomyomas datasets (GSE12814 and GSE23112) revealed the regulation of several biological processes as a significantly enriched BP, while membrane and binding processes were detected as a significantly enriched CC and MF, respectively (Figure 3).

## 4. Discussion

Uterine pathologies impact women’s health and quality of life considerably. In recent years, the advent of omics technologies has facilitated a comprehensive exploration of molecular patterns associated with some of the most common gynecological conditions [57,58,59,60]. Microarray technology emerged about three decades ago with the aim of studying whole gene expression profiles, and the analysis of the amount of data derived from the application of this powerful tool has provided unprecedented insights into the discovery of dysregulated molecular pathways implicated in disease pathogenesis [61,62]. In the present study, we analyzed data from six datasets generated from the application of Affymetrix microarray devices: two datasets from endometrial cancer, two datasets from endometriosis, and two datasets from uterine leiomyomas.

The raw data were downloaded from the GEO public repository, and the gene expression files were preprocessed, quality-controlled, and normalized. For the detection of DEGs, two strategies were adopted: (i) a traditional approach based on the use of classical statistical *t*-tests, and (ii) an alternative approach using the CASh method [49]. We were not able to detect, in general, DEGs using traditional approaches, while the use of the CASh method revealed a number of statistically significant genes in the six datasets analyzed. The *t*-test selects genes according to their differential expression between the two study conditions at an individual level. Thus, genes are considered significant when its *p*-value is below an established threshold (0.05 adj. *p*-value in our study). On the other hand, the CASh method considers not only the expression of each gene under two conditions but the contribution of those genes over all possible permutations of genes, using the Shapley value to measure this contribution. The CASh method evaluates the gene expression as a co-operative game, where the Shapley value quantifies the importance of each gene based on its contribution across all possible subsets of genes. This holistic evaluation helps mitigate the impact of confounding variables by considering the overall gene network rather than isolated gene expressions. However, a current limitation of CASh is that it does not explicitly account for potential confounding effects. Addressing these confounding variables in future applications needs to be studied further [49,51,52,53]. In brief, CASh offers a more nuanced understanding of gene interactions and their collective impact on disease pathophysiology.

Interestingly, the functional enrichment analysis of the DEGs detected using the CASh method confirmed previous findings on the molecular bases of the uterine pathologies analyzed in our study. Some processes related to cell cycle and proliferation events have been shown to be significantly dysregulated in our sets of DEGs. Given the nature of endometrial cancer and endometriosis, it is plausible to believe that alterations at gene expression levels in some genes involved in these proliferative pathways may contribute to the phenotype of these diseases, as it has been previously proposed [63,64]. Further, a possible role of the degradation and remodeling of the extracellular matrix in endometriosis datasets has been revealed in our study. Endometriotic tissues have been shown to be significantly associated to extracellular matrix reorganization in some studies, which may explain some of the molecular mechanisms underlying the progression of the disease [65,66,67,68]. Regarding uterine leiomyomas, we were able to detect some significantly enriched biological processes that have been previously reported in association with the disease such as hormone secretion and cell signaling [69].

Our preliminary results underscore the potential of CASh as a valuable tool for analyzing microarray data. Further extensive research, including validation studies on larger cohorts and functional assays, is warranted to confirm the robustness and clinical relevance of the identified molecular signatures.

## 5. Conclusions

This study underscores the utility of the Comparative Analysis of Shapley value (CASh) in uncovering nuanced genetic insights into common uterine pathologies such as endometriosis, uterine leiomyomas, and endometrial cancer. Our findings not only enhance the existing understanding of the molecular underpinnings of these conditions but also pave the way for innovative diagnostic and therapeutic strategies. The application of CASh has demonstrated a significant improvement in identifying differentially expressed genes, which are often overlooked by traditional statistical methods.

Looking forward, the integration of CASh with other omics technologies, such as proteomics and metabolomics, could provide a more comprehensive understanding of the pathophysiological landscapes of uterine diseases. Such integrative approaches are anticipated to facilitate the development of multi-marker panels that could improve the specificity and sensitivity of diagnostic tools. Additionally, longitudinal studies employing CASh could monitor disease progression and response to treatment, providing valuable insights into the dynamic nature of gene expression changes associated with disease states.

Collaborations across interdisciplinary teams comprising geneticists, gynecologists, oncologists, and bioinformaticians will be essential in order to harness the full potential of these findings. Such collaborations could lead to large-scale studies that validate and refine the predictive power of identified gene signatures and explore their utility in clinical settings.

Ultimately, the goal of this research is to contribute to precision medicine approaches that tailor preventive, diagnostic, and therapeutic strategies to the individual genetic profiles of patients suffering from uterine pathologies. By improving our understanding of the genetic basis of these diseases, we aim to enhance patient outcomes through more targeted and effective interventions, reducing the burden these conditions place on women globally.

## 6. Limitations of the Study

While the application of the Comparative Analysis of Shapley values (CASh) has provided valuable insights into the transcriptomic profiles of common uterine pathologies, this study is not without limitations. First, the inherent complexity of microarray data, including issues related to noise, batch effects, and variability in sample quality, can impact the accuracy of gene expression analysis. Despite rigorous preprocessing and normalization procedures, these factors might still influence the results and interpretations of our findings.

Second, the study relies on datasets obtained from public repositories, which may contain biases due to the methods of data collection, patient selection, and experimental design employed by the original researchers. The generalizability of our results to other populations or to clinical settings may, therefore, be limited.

Additionally, the computational intensity of CASh, particularly when applied to large datasets, poses significant challenges. The method requires substantial computational resources, and the interpretation of Shapley values can be complex, potentially limiting its utility in routine clinical practice without further simplification and validation.

Furthermore, while CASh provides a robust framework for identifying key genes, it does not account for potential post-transcriptional modifications or protein-level interactions, which are crucial for a full understanding of the molecular mechanisms underlying these pathologies. Integrating our approach with proteomic and metabolomic data could, therefore, enhance the depth of our findings.

Finally, our study design does not include the experimental validation of the identified differentially expressed genes. Future studies involving functional assays are necessary in order to confirm the roles of these genes in disease mechanisms and their potential as therapeutic targets.

## Figures and Tables

**Table 1:** Summary of Gene Expression Omnibus (GEO) datasets analyzed in our study. For each study, number and description of samples are shown.

Phenotype Group	Dataset ID	No. of Samples	Description of Samples
Endometrial cancer	GSE36389	16	Endometrial cancer (n = 10) vs. controls (n = 6)
	GSE63678	11	Endometrial carcinoma (n = 6) vs. controls (n = 5)
Endometriosis	GSE7846	9	Endometriosis (n = 4) vs. controls (n = 5)
	GSE17504	11	Endometriosis (n = 5) vs. controls (n = 6)
Uterine leiomyomas	GSE12814	14	Uterine leiomyoma (n = 5) vs. controls (n = 9)
	GSE23112	7	Uterine leiomyoma (n = 3) vs. controls (n = 4)

**Table 2:** Number of differentially expressed genes (DEGs) detected after the analysis using conventional techniques based on Welch’s *t*-test and Empirical Bayes (EBayes), and alternative approaches rooted in Comparative Analysis of Shapley value (CASh) method with cutoff raw *p*-values of 0.01 or 0.05, respectively. FDR-corrected *p*-values are included where indicated.

Dataset ID	Welch’s *t*-Test	EBayes FDR < 0.01	EBayes FDR < 0.05	CASh 0.05 FDR < 0.05	CASh 0.01	CASh 0.05
GSE36389	0	0	0	0	33 (18 ↑, 15 ↓)	115 (67 ↑, 48 ↓)
GSE63678	0	0	358	33 (15 ↑, 18 ↓)	496 (213 ↑, 283 ↓)	935 (456 ↑, 479 ↓)
GSE7846	0	0	0	140 (81↑, 59 ↓)	71 (39 ↑, 32 ↓)	333 (194 ↑, 139 ↓)
GSE17504	0	0	0	17 (12 ↑, 5 ↓)	17 (9 ↑, 8 ↓)	83 (49 ↑, 34 ↓)
GSE12814	0	1	75	0	22 (14 ↑, 8 ↓)	91 (40 ↑, 51 ↓)
GSE23112	0	0	0	16 (6 ↑, 7 ↓)	6 (5 ↑, 1 ↓)	33 (23 ↑, 10 ↓)

↑ and ↓ symbols indicate up- (FC > 2) and down-regulated (FC < −2) genes, respectively.

## Data Availability

The microarray data were obtained from Gene Expression Omnibus (GEO) database (https://www.ncbi.nlm.nih.gov/geo, accessed on 28 May 2024) as stated above. The custom scripts used for data analysis are deposited in the public repository Zenodo and are available through https://zenodo.org/records/11222132, accessed on 28 May 2024.

## Supplementary Materials

The following supporting information can be downloaded at: https://www.mdpi.com/article/10.3390/genes15060723/s1, Table S1: Technical description of the datasets analyzed in the present study; Table S2: Differentially expressed genes obtained for each dataset after statistical analyses; Table S3: Functional enrichment analysis of the differentially expressed genes obtained in each dataset through the application of Comparative Analysis of Shapley values with raw *p*-values 0.01 and 0.05; Figure S1: Exploratory analysis results: Principal Component Analysis (PCA) showing the distribution of gene expression patterns across all the datasets; Figure S2: Exploratory analysis results: side-by-side volcano plots showing the comparison between the different tests statistics applied to each dataset; Figure S3: Exploratory analysis results: heatmap showing the distribution of the differentially expressed genes identified by different methods across all the datasets.
