# Supplementary material for: Comparative Analysis of Shapley Values Enhances Transcriptomics Insights across Some Common Uterine Pathologies
Source: Genes (Basel). 2024 Jun 1;15(6):723. doi: 10.3390/genes15060723 (PMC11203383; doi:10.3390/genes15060723)
Supplement: Supplementary file 1 [file genes-15-00723-s001.zip › SupplementaryFigure_1.pptx]

## Slide 1
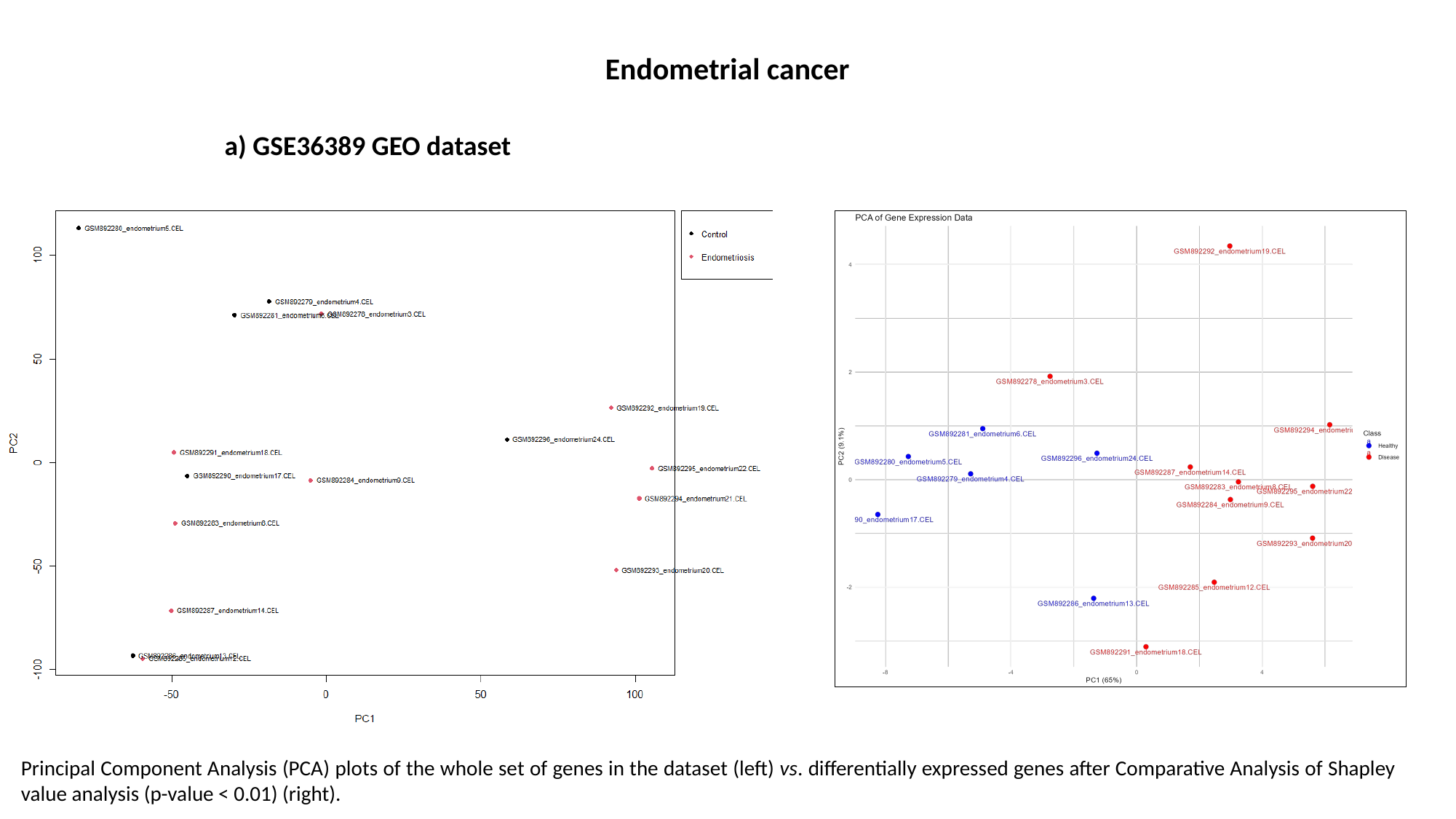

Endometrial cancer
a) GSE36389 GEO dataset
Principal Component Analysis (PCA) plots of the whole set of genes in the dataset (left) vs. differentially expressed genes after Comparative Analysis of Shapley value analysis (p-value < 0.01) (right).

## Slide 2
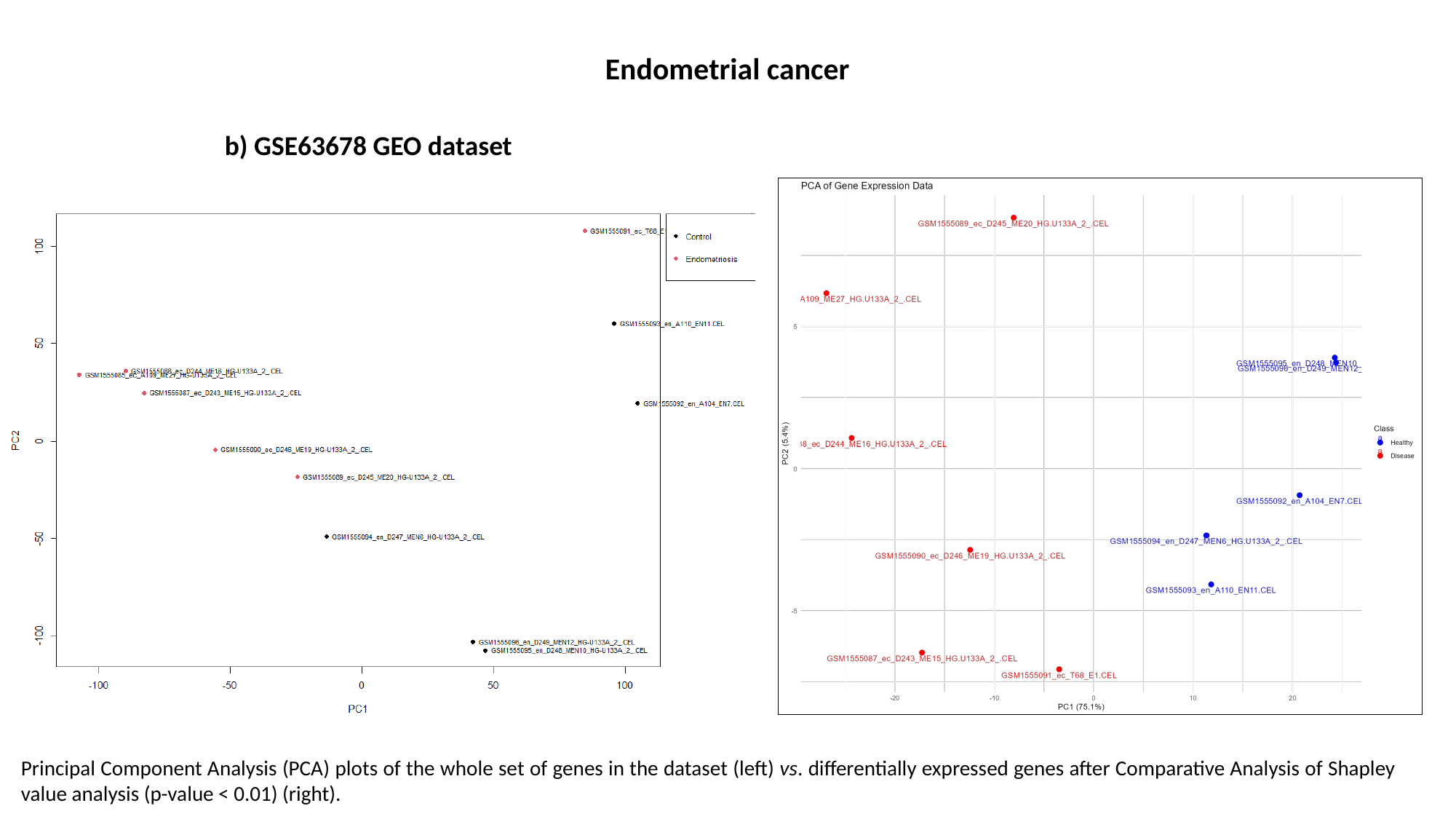

Endometrial cancer
b) GSE63678 GEO dataset
Principal Component Analysis (PCA) plots of the whole set of genes in the dataset (left) vs. differentially expressed genes after Comparative Analysis of Shapley value analysis (p-value < 0.01) (right).

## Slide 3
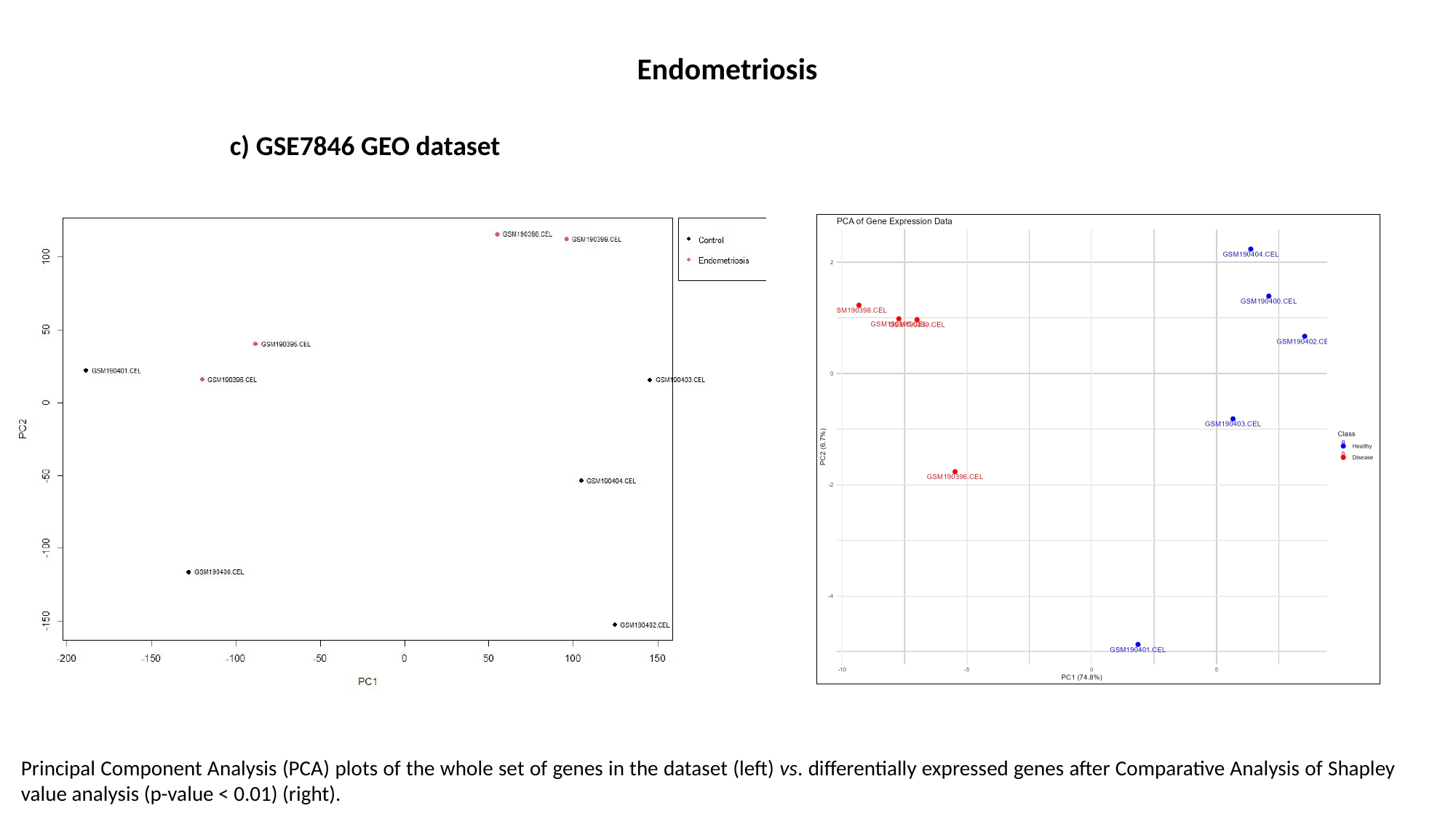

Endometriosis
c) GSE7846 GEO dataset
Principal Component Analysis (PCA) plots of the whole set of genes in the dataset (left) vs. differentially expressed genes after Comparative Analysis of Shapley value analysis (p-value < 0.01) (right).

## Slide 4
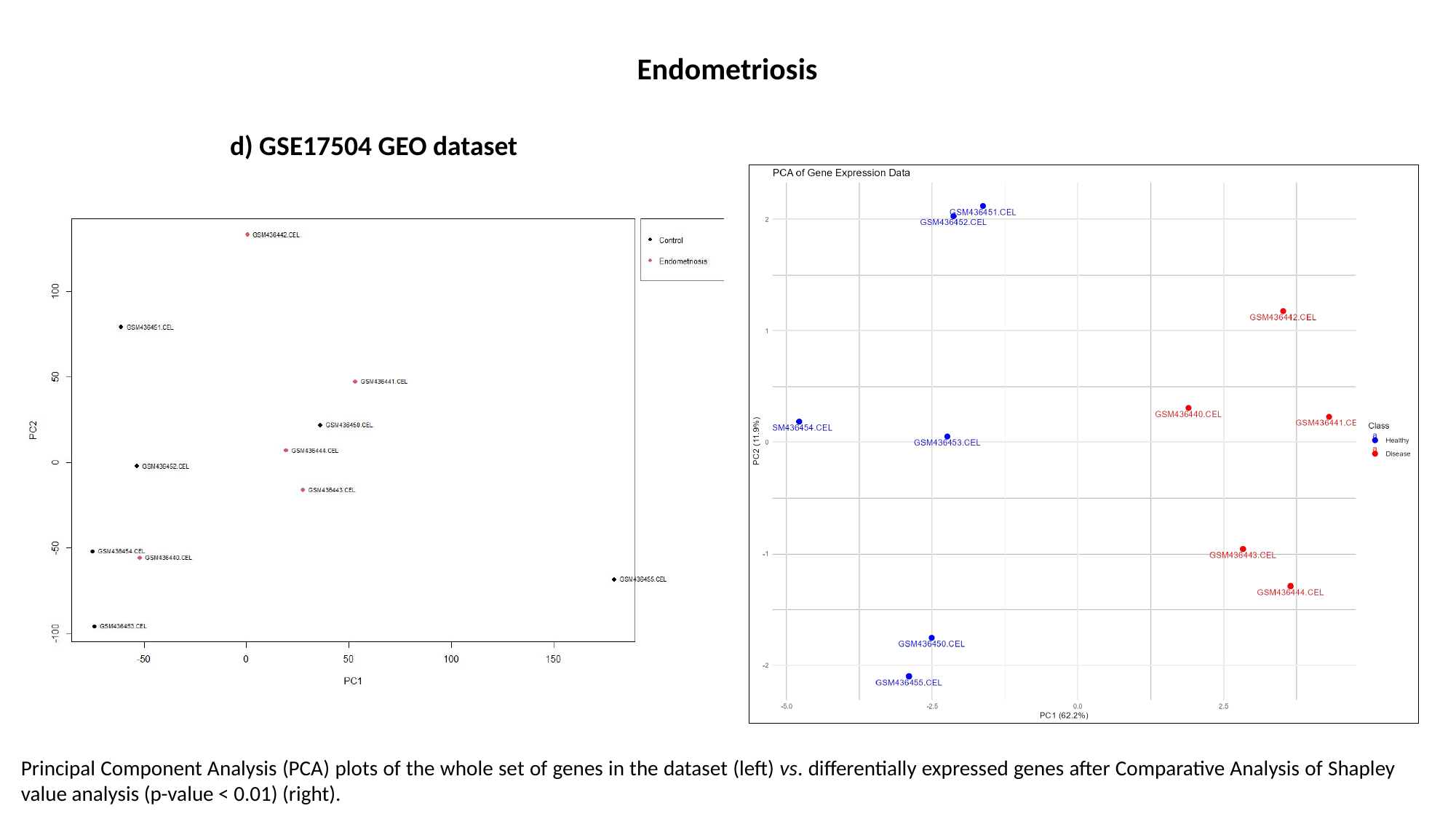

Endometriosis
d) GSE17504 GEO dataset
Principal Component Analysis (PCA) plots of the whole set of genes in the dataset (left) vs. differentially expressed genes after Comparative Analysis of Shapley value analysis (p-value < 0.01) (right).

## Slide 5
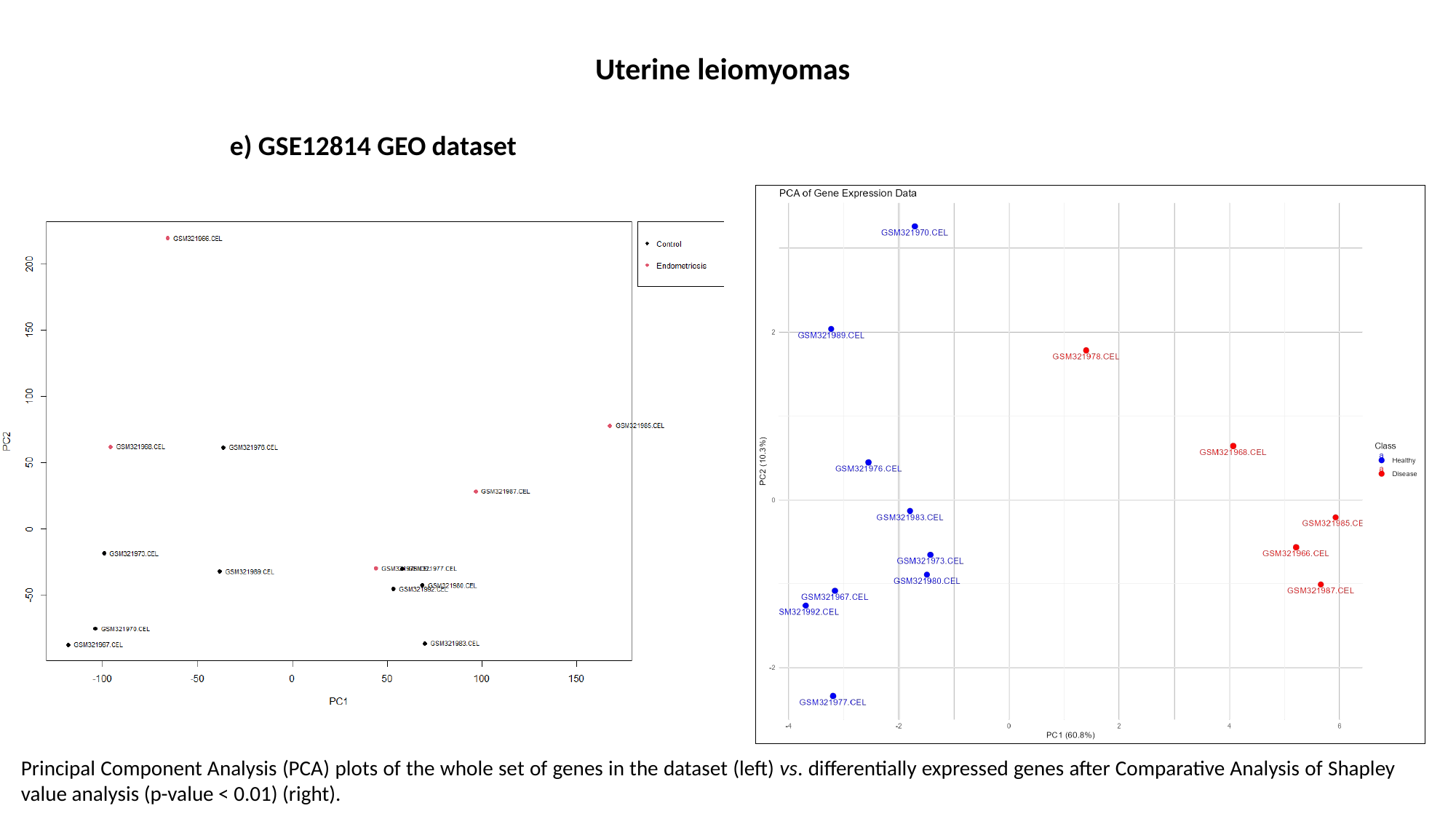

Uterine leiomyomas
e) GSE12814 GEO dataset
Principal Component Analysis (PCA) plots of the whole set of genes in the dataset (left) vs. differentially expressed genes after Comparative Analysis of Shapley value analysis (p-value < 0.01) (right).

## Slide 6
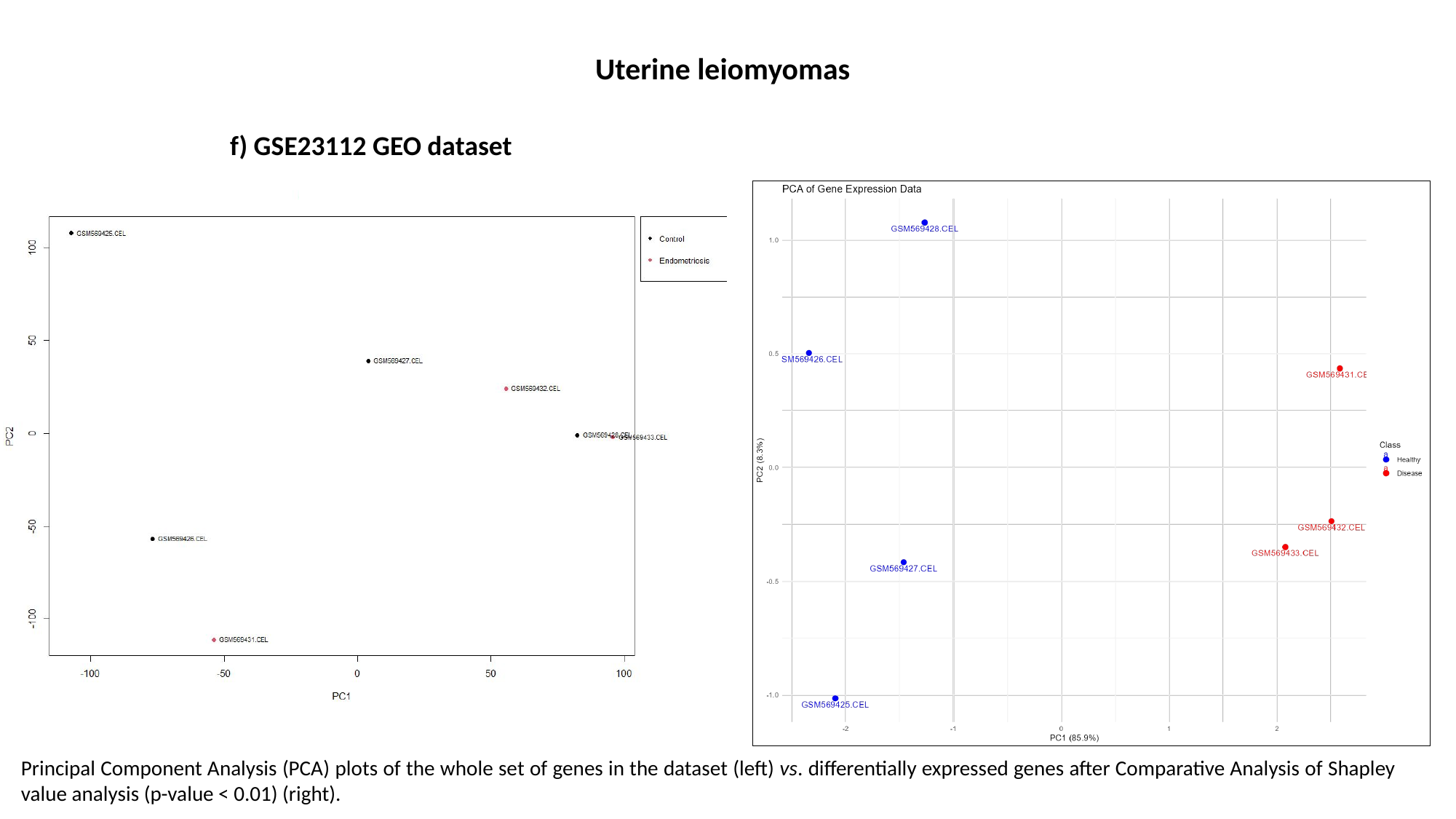

Uterine leiomyomas
f) GSE23112 GEO dataset
Principal Component Analysis (PCA) plots of the whole set of genes in the dataset (left) vs. differentially expressed genes after Comparative Analysis of Shapley value analysis (p-value < 0.01) (right).
