# Supplementary material for: Comparative Analysis of Shapley Values Enhances Transcriptomics Insights across Some Common Uterine Pathologies
Source: Genes (Basel). 2024 Jun 1;15(6):723. doi: 10.3390/genes15060723 (PMC11203383; doi:10.3390/genes15060723)
Supplement: Supplementary file 1 [file genes-15-00723-s001.zip › SupplementaryFigure_2.pptx]

## Slide 1
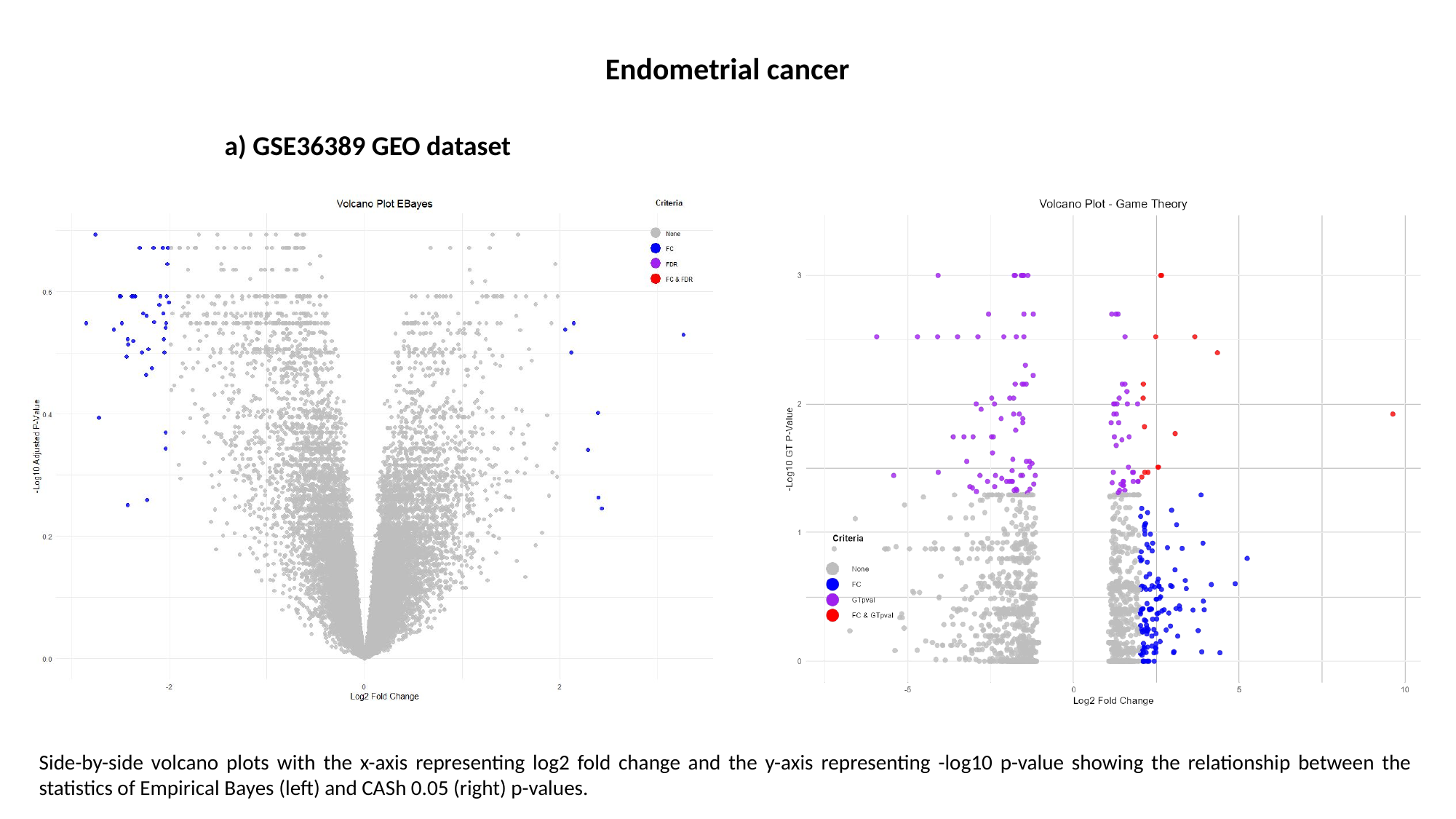

Endometrial cancer
a) GSE36389 GEO dataset
Side-by-side volcano plots with the x-axis representing log2 fold change and the y-axis representing -log10 p-value showing the relationship between the statistics of Empirical Bayes (left) and CASh 0.05 (right) p-values.

## Slide 2
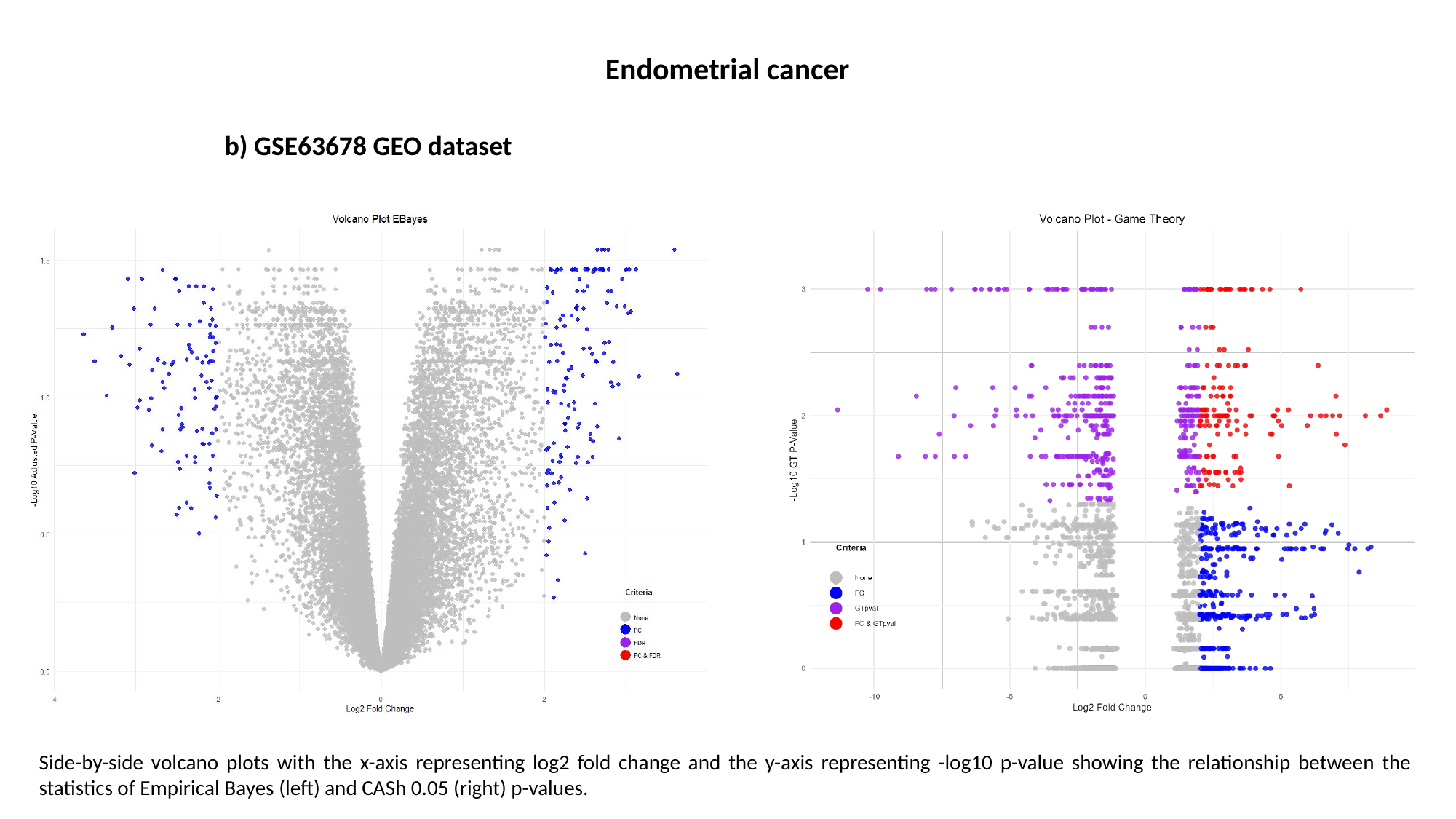

Endometrial cancer
b) GSE63678 GEO dataset
Side-by-side volcano plots with the x-axis representing log2 fold change and the y-axis representing -log10 p-value showing the relationship between the statistics of Empirical Bayes (left) and CASh 0.05 (right) p-values.

## Slide 3
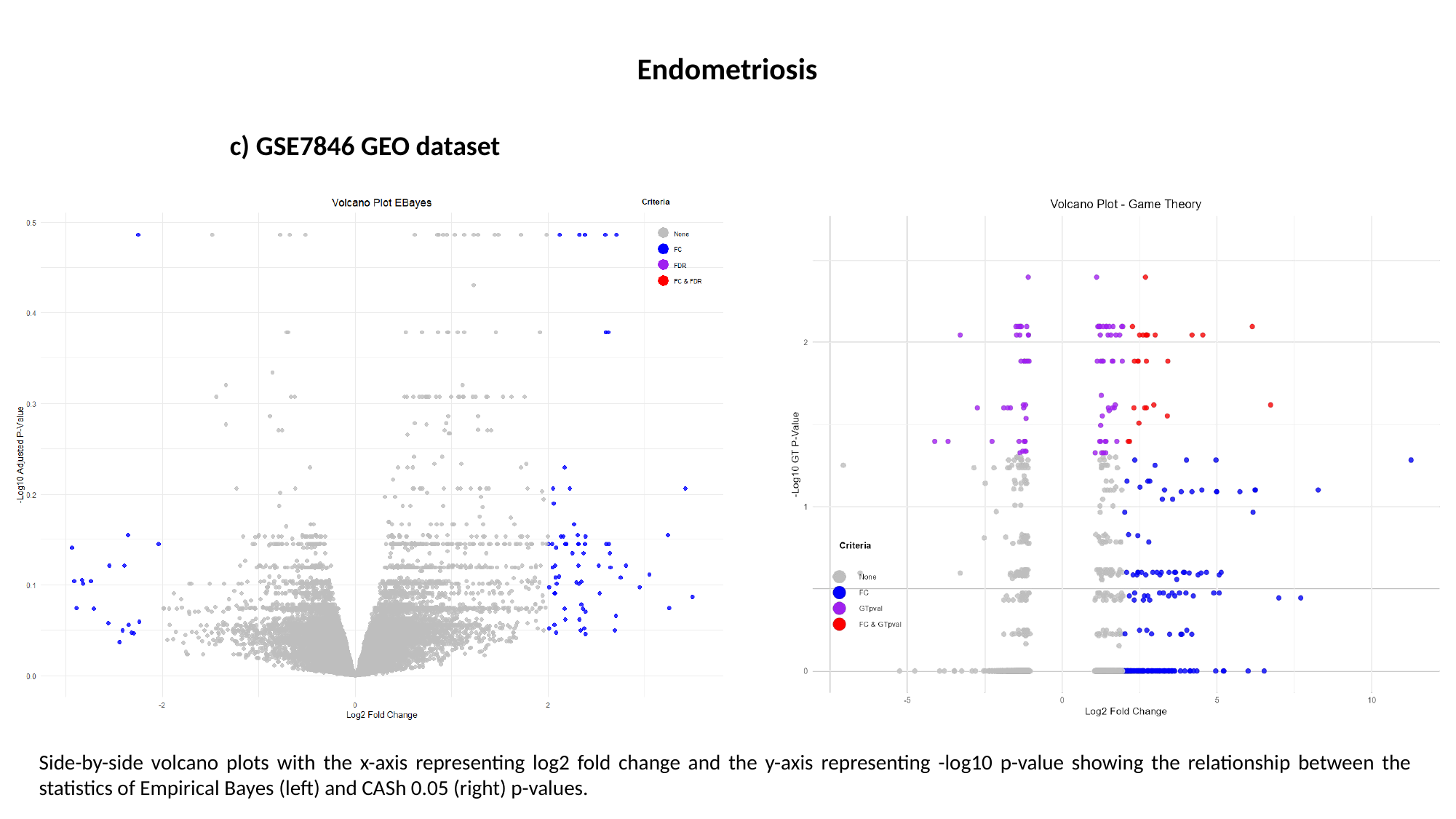

Endometriosis
c) GSE7846 GEO dataset
Side-by-side volcano plots with the x-axis representing log2 fold change and the y-axis representing -log10 p-value showing the relationship between the statistics of Empirical Bayes (left) and CASh 0.05 (right) p-values.

## Slide 4
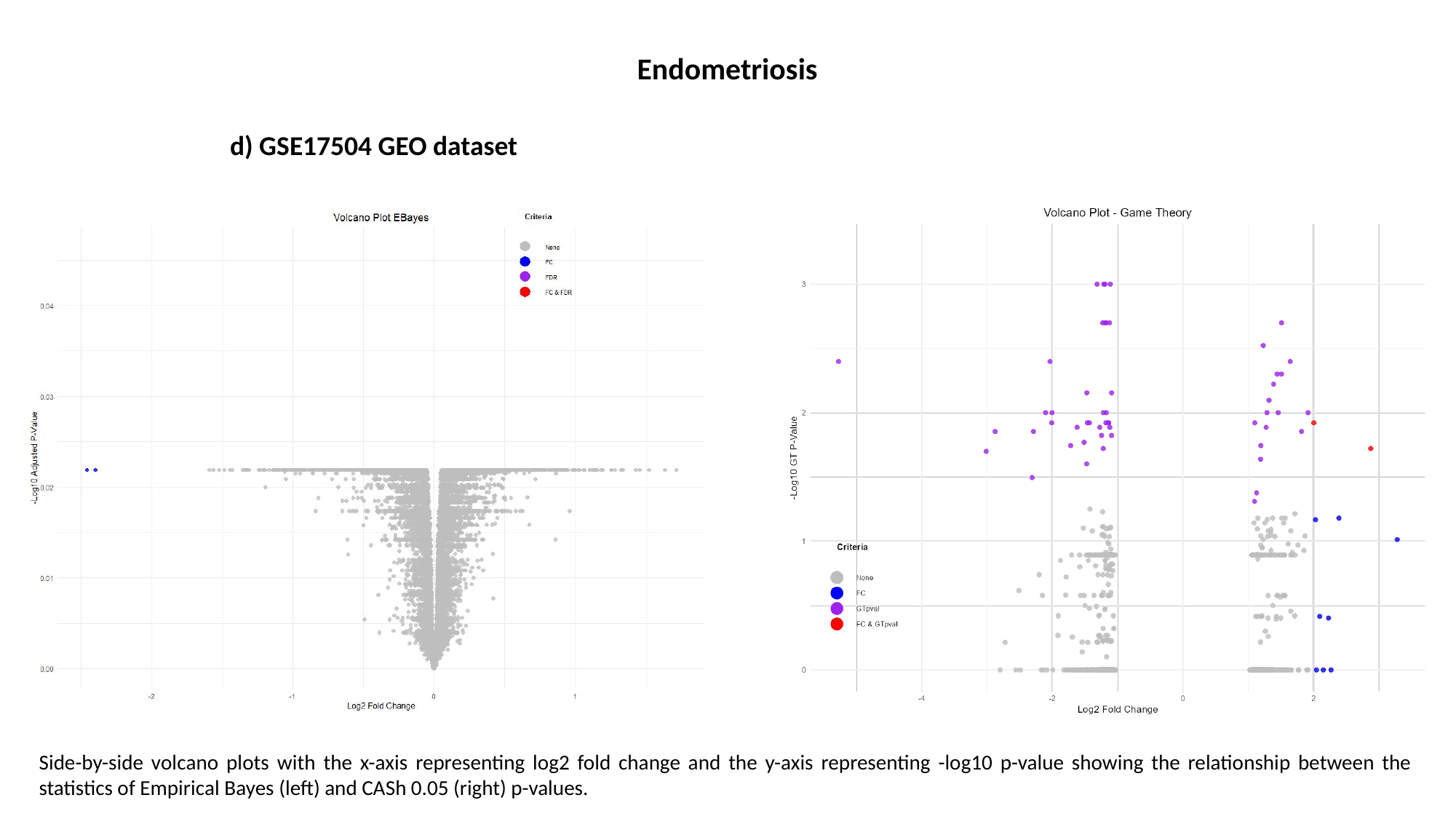

Endometriosis
d) GSE17504 GEO dataset
Side-by-side volcano plots with the x-axis representing log2 fold change and the y-axis representing -log10 p-value showing the relationship between the statistics of Empirical Bayes (left) and CASh 0.05 (right) p-values.

## Slide 5
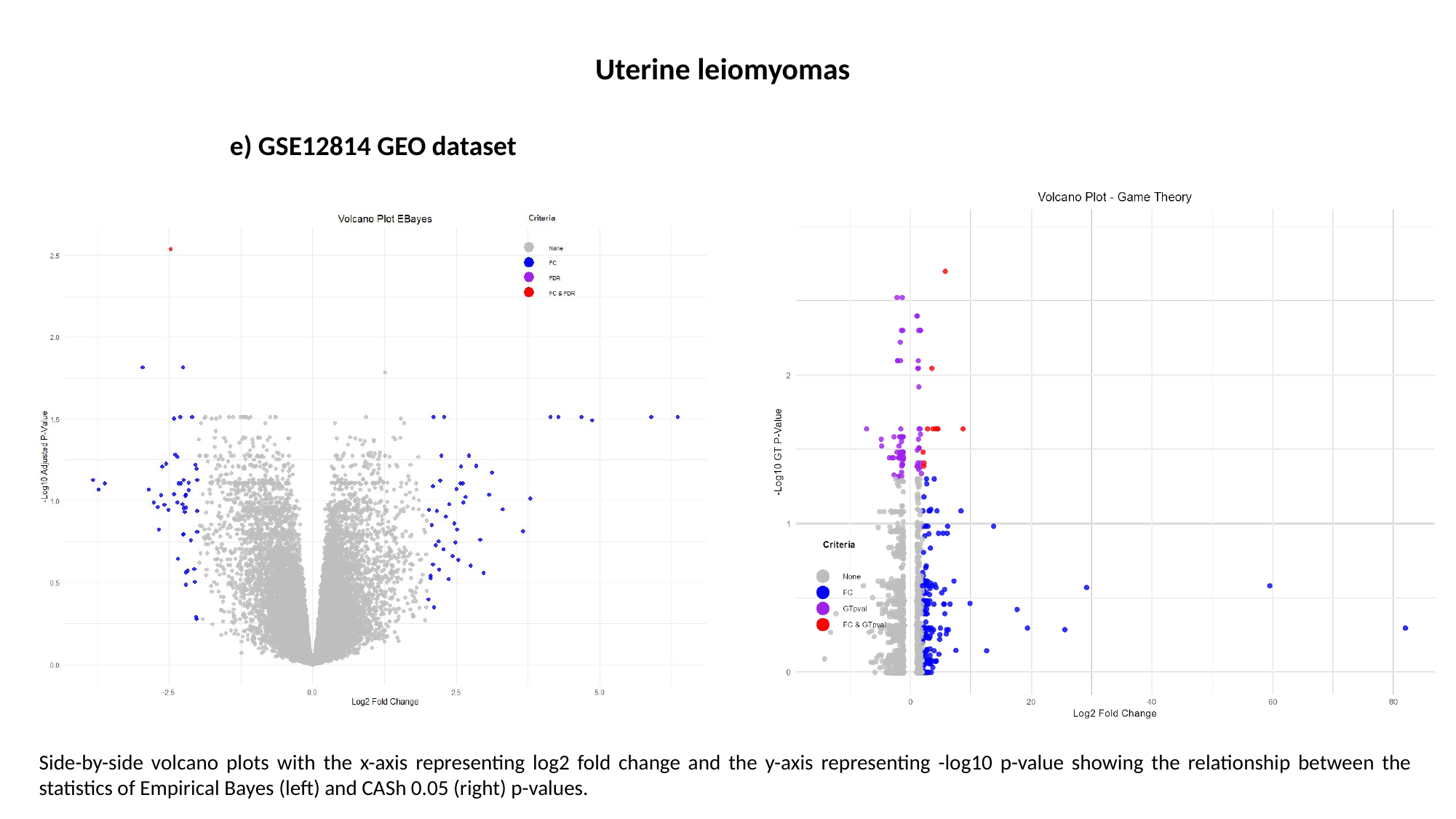

Uterine leiomyomas
e) GSE12814 GEO dataset
Side-by-side volcano plots with the x-axis representing log2 fold change and the y-axis representing -log10 p-value showing the relationship between the statistics of Empirical Bayes (left) and CASh 0.05 (right) p-values.

## Slide 6
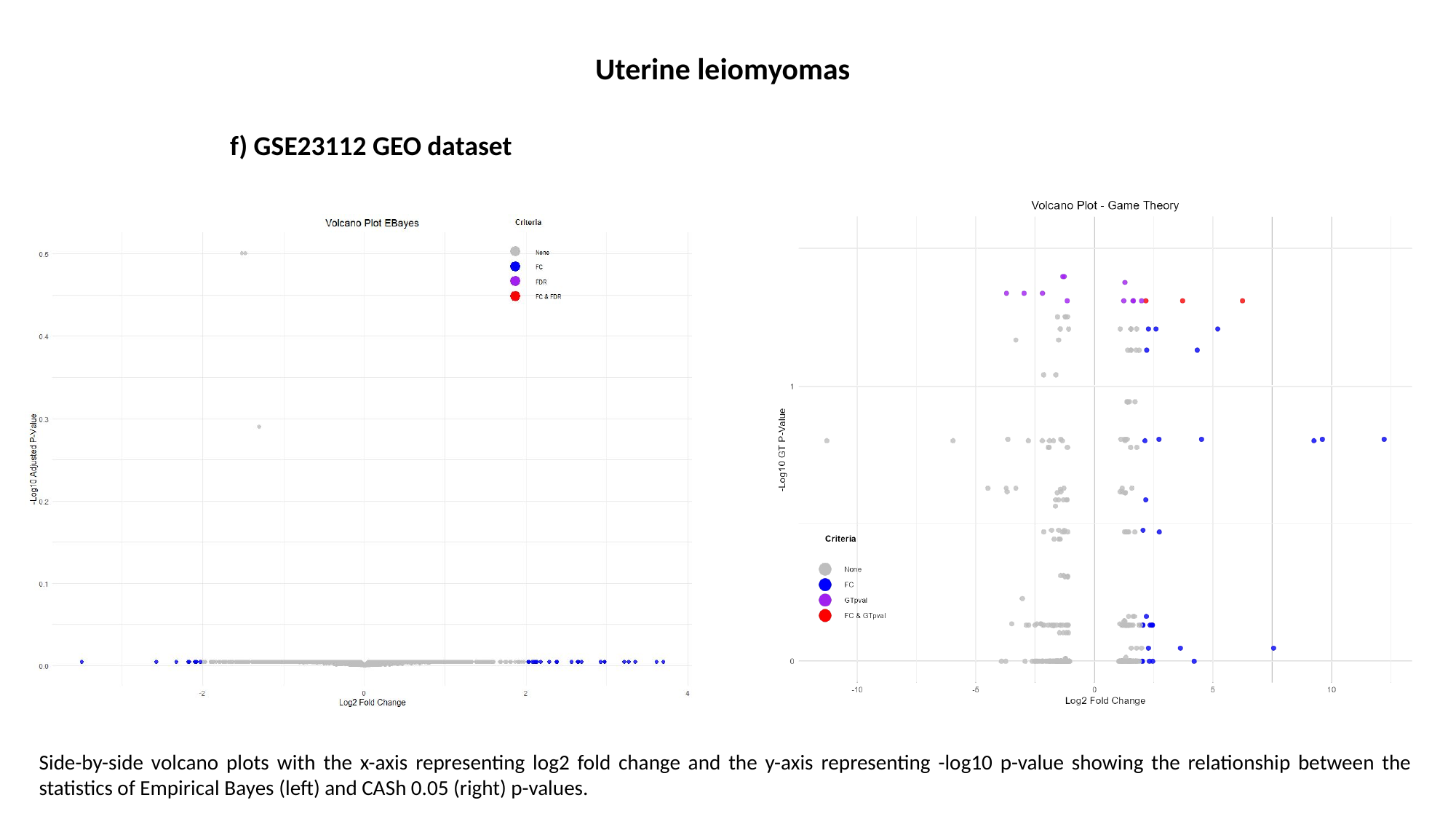

Uterine leiomyomas
f) GSE23112 GEO dataset
Side-by-side volcano plots with the x-axis representing log2 fold change and the y-axis representing -log10 p-value showing the relationship between the statistics of Empirical Bayes (left) and CASh 0.05 (right) p-values.
